# Salvage Plasmapheresis for Post-hepatectomy Liver Failure

**DOI:** 10.7759/cureus.884

**Published:** 2016-11-17

**Authors:** Faisal Inayat, Qulsoom Hussain, Syed H Tasleem, Sajid Farooq, Abu Hurairah

**Affiliations:** 1 Department of Medicine, New York-Presbyterian Hospital, Weill Cornell Medical College, New York City, NY, USA; 2 Department of Medicine, Shifa International Hospital, Shifa College of Medicine, Islamabad, Pakistan; 3 Department of Hepatology and Multiorgan Transplant, Beaumont Hospital, Royal Oak, MI, USA; 4 Department of Pulmonary and Critical Care Medicine, Truman Medical Center, University of Missouri School of Medicine, Kansas City, MO, USA; 5 Division of Gastroenterology, Department of Medicine, SUNY Downstate Medical Center, Brooklyn, NY, USA

**Keywords:** post-hepatectomy, liver failure, management, plasmapheresis

## Abstract

In the latest decades, an important change has been registered in liver surgery related to the progress of surgical techniques, critical care, and postoperative treatment, allowing a sharp decrease in mortality and morbidity. However, management of post-hepatectomy liver failure (PHLF) still remains a challenge and no supportive treatment has been found to be generally effective. The present study is a reappraisal of plasmapheresis as a potential supportive measure in patients with PHLF following major liver resection.

## Introduction

Post-hepatectomy liver failure (PHLF) syndrome is a feared complication of major liver resection leading to liver failure with resultant high mortality. Treatment options are limited and mostly supportive [1]. Herein, we describe a case of PHLF after extended right hepatectomy for hepatocellular carcinoma in a non-cirrhotic liver, where plasmapheresis was used to salvage the remnant liver. An informed consent statement was obtained from the patient for this study.

## Case presentation

**Figure 1:**
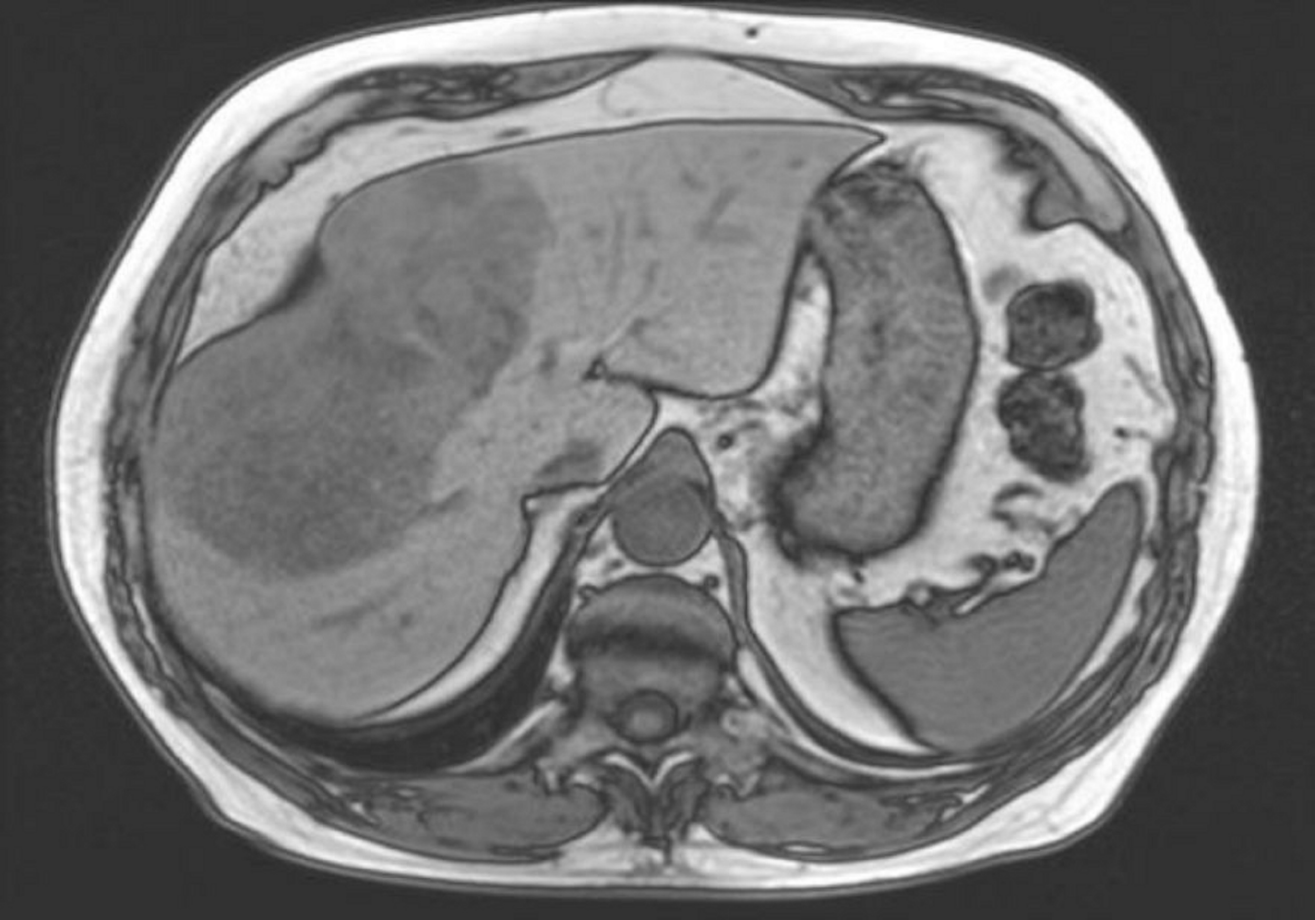
Right lobe hepatocellular carcinoma pre-resection

The patient was a 76-year-old male with biopsy-proven hepatocellular carcinoma in a non-cirrhotic liver. Magnetic resonance imaging (MRI) demonstrated a 15.9 cm heterogeneously enhancing hepatic mass in segments 4a/b, 5 and 8 (LI-RAD 4B) without any evidence of portal/hepatic vein thrombosis or lymph node involvement (Figure 1).

Further imaging with computerized tomography (CT) of the chest and positron emission tomography (PET) scan ruled out metastatic disease. Liver biopsy of the uninvolved parenchyma revealed no significant fibrosis.

**Figure 2:**
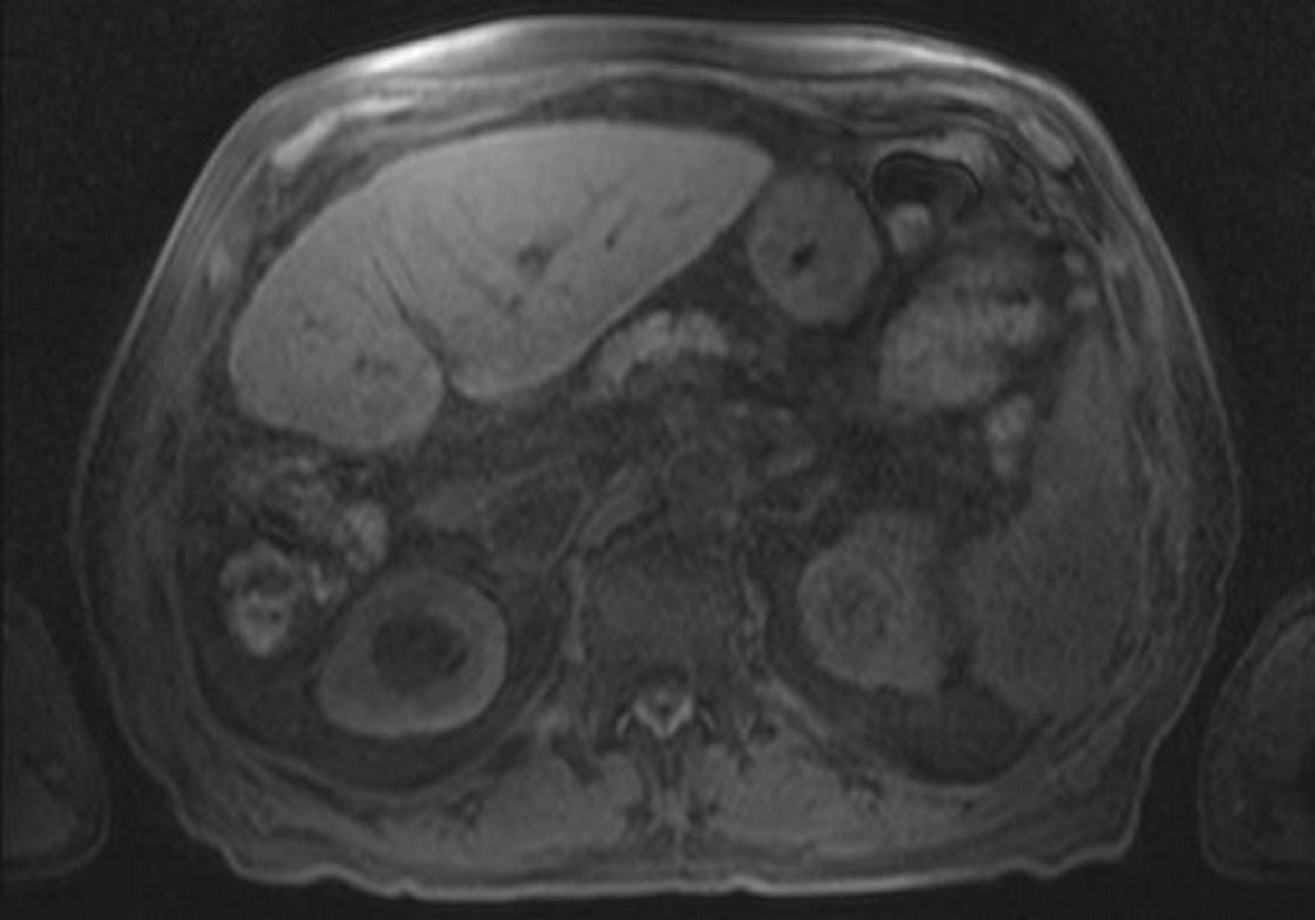
Remnant liver post-resection

The patient underwent exploratory laparotomy with extended right hepatectomy and cholecystectomy (Figure 2).

A remnant liver volume of >30% was achieved. In the immediate postoperative period, he developed liver failure (ascites, peak total bilirubin 31, INR 2.9 and grade I/II hepatic encephalopathy) due to PHLF. After total bilirubin peaked at 31, salvage plasmapheresis was started. He received 17 consecutive treatments of plasmapheresis with marked clinical improvement and overall downtrending of his total bilirubin and international normalized ratio (INR).

The patient gradually recovered after a protracted hospital stay, which was further complicated by acute kidney failure (requiring hemodialysis), portal vein thrombosis and multiple infections (multi-drug resistant (MDR) Klebsiella pneumonia bacteremia and MDR Enterobacter cloacae in the surgical bed). He was discharged after seven months in a good condition. The patient was tumor free, with completely normal liver function at the 15-month follow-up.

## Discussion

Hepatic resections are among some of the most complex operative interventions performed and are fraught with risk and the potential for complications. Mortality rates after major hepatic resection have been reported to be as high as 30% [2]. PHLF is a clinical syndrome that represents the major source of morbidity and mortality after liver resection. Despite great improvements in outcomes after major hepatectomy, PHLF remains one of the most serious complications of major liver resection and occurs in up to 10% of the cases [2].

PHLF is usually marked by coagulopathy, hyperbilirubinemia and hepatic encephalopathy which occurs after extended hepatectomy. Its pathophysiology is mediated by postoperative portal hyperperfusion (into the liver remnant) which leads to transient portal hypertension, sinusoidal constriction and endothelial injury [1]. Morbidity and mortality is intimately related to failure of remnant liver and/or infection.

Risk factors of the development of PHLF includes not only the size of the remnant liver but also underlying cirrhosis, steatosis, advanced age, protein-calorie malnutrition, concomitant diabetes, existence of preoperative portal hypertension and preoperative Mayo End-stage Liver Disease (MELD) score of >31 [1-2]. Infection complicates the course either as a precipitant or during later stages due to the reduced phagocytosis capacity of the hepatic reticuloendothelial system [2]. Diminished hepatic clearance of bacteria might enhance the susceptibility for the development of infections.

Management of PHLF hinges first on its prevention. In patients identified as high-risk by preoperative evaluation, consideration should be given to techniques to minimize the risk of PHLF. One such technique is portal vein embolization (PVE), which manipulates portal blood flow by embolizing portal branches in the liver to be resected, directing blood flow to the intended remnant liver and thereby inducing hypertrophy of the remnant liver before major hepatectomy [3]. In some circumstances, a surgeon may employ portal vein ligation (PVL) or the associating liver partition and portal vein ligation for staged hepatectomy (ALPPS) procedure [1-3].

Goal-directed therapy should be provided in patients with PHLF for circulatory disturbances, renal and ventilatory dysfunction, coagulopathy, malnutrition and hepatic encephalopathy [1-2]. As there seems to be a strong link between infection and post-resection liver failure, frequent cultures for bacteria and fungi are essential. The most effective treatment for PHLF is liver transplantation, but this is typically reserved for patients who have failed all other supportive therapies [3-4]. The support therapies for PHLF include infusion of albumin, fibrinogen, fresh frozen plasma, blood transfusion, and initiation of nutritional supplementation [4].

Plasmapheresis, a method of plasma exchange to replace fresh plasma after separation from blood, may be employed in patients with PHLF [2]. Although the role of plasmapheresis in liver failure is not clearly established yet, encouraging reports have addressed its efficacy in the setting of resections for small-for-size syndrome after live donor liver transplantation and acute liver failure. However, to our knowledge, only few case reports described plasmapheresis as the successful salvage strategy for PHLF [5-6].

In their case report, Hwing S, et al. highlighted that a rapid correction of serum cholesterol level and prothrombin time was achieved after only two sessions of plasmapheresis, which was indicative of the rapid recovery of hepatic synthetic function [5]. Sotiropoulos GC, et al. reported the successful treatment of postoperative liver failure after hepatic resection for colorectal liver metastases using plasmapheresis [6].

In the present patient, after plasmapheresis, the improvement in hepatic synthetic and metabolic function was clinically evident and eventually resulted in normalization of total bilirubin and INR. It was previously thought that liver support from plasmapheresis was too weak to reverse the sequence of definite hepatic failure [5]. However, our experience with this patient suggests that in patients with PHLF plasmapheresis can trigger spontaneous recovery.

## Conclusions

Despite great improvements in morbidity and mortality, liver surgery continues to demand excellent clinical judgment in the postoperative period. There is limited literature to support the use of plasmapheresis to decrease portal hyperperfusion and bilirubin after extensive hepatectomy. The present study demonstrates that plasmapheresis may be life-saving and should be considered as a viable supportive treatment in patients with PHLF.

## Appendices

The patient agreed to participate and was explained the nature and objectives of this study, and informed consent was formally obtained from the patient. No reference to the patient's identity was made at any stage during data analysis or in the report.
